# Supplementary material for: The neglected epidemic—Risk factors associated with road traffic injuries in Mozambique: Results of the 2016 INCOMAS study
Source: PLOS Glob Public Health. 2022 Feb 28;2(2):e0000163. doi: 10.1371/journal.pgph.0000163 (PMC10021512; doi:10.1371/journal.pgph.0000163)
Supplement: Attachment — Submitted filename: Response to reviewers.docx [file pgph.0000163.s002.docx]

12/8/2021

Dear reviewers and Plos Global Health Editors,

Thanks very much for the comments and suggestions. Please see attached our detailed responses to your questions.

Responses to editorial comments

1. Please include additional information regarding the survey or questionnaire used in the study and ensure that you have provided sufficient details that others could replicate the analyses. For instance, if you developed a questionnaire as part of this study and it is not under a copyright more restrictive than CC-BY, please include a copy, in both the original language and English, as Supporting Information.

**The INCOMAS questionnaire items used in this study are attached in English and Portuguese. (xls file name: INCOMAS Injury only)**

2. Please provide us with a direct link to the base layer of the map used in Figure 2 and ensure this location is also included in the figure legend.

**We used United Nations maps for the Mozambique map, and the Word Bank for Mozambique roads. The maps were created in R using the packages tidyverse (ggplot2), sf, and Maps. To our best knowledge all the materials used are open source**

**Mozambique Roads** [**https://datacatalog.worldbank.org/dataset/mozambique-roads**](https://datacatalog.worldbank.org/dataset/mozambique-roads)

**Mozambique map** [**https://data.humdata.org/dataset/mozambique-administrative-levels-0-3**](https://data.humdata.org/dataset/mozambique-administrative-levels-0-3)

3. Please note that, because all PLOS articles are published under a CC BY license (creativecommons.org/licenses/by/4.0/), we cannot publish proprietary maps such as Google Maps, Mapquest or other copyrighted maps. If your map was obtained from a copyrighted source please amend the figure so that the base map used is from an openly available source.

**Please see our response to point 2**

4. During our internal checks, the in-house editorial staff noted that you conducted research or obtained samples in another country. Please check the relevant national regulations and laws applying to foreign researchers and state whether you obtained the required permits and approvals. Please address this in your ethics statement in both the manuscript and submission information. In addition, please ensure that you have suitably acknowledged the contributions of any local collaborators involved in this work in your authorship list and/or Acknowledgements. Authorship criteria is based on the International Committee of Medical Journal Editors (ICMJE) Uniform Requirements for Manuscripts Submitted to Biomedical Journals - for further information please see here: [https://journals.plos.org/plosone/s/authorship](https://eur03.safelinks.protection.outlook.com/?url=https%3A%2F%2Fjournals.plos.org%2Fplosone%2Fs%2Fauthorship&data=04%7C01%7Caperaltasantos%40dgs.min-saude.pt%7Cd6e78db0eb174b26804808d97d53bbc7%7C22c84608f01d46c5802463cc962e5f51%7C0%7C0%7C637678620873923470%7CUnknown%7CTWFpbGZsb3d8eyJWIjoiMC4wLjAwMDAiLCJQIjoiV2luMzIiLCJBTiI6Ik1haWwiLCJXVCI6Mn0%3D%7C1000&sdata=KVcufp2nqRY5TRdqCUnhpCr0wigmvVDARKIME9yQqCY%3D&reserved=0).

**See updated information regarding the ethics review on lines 151-155.**

***“This study was approved by the Institutional Review Board of the National Institute of Health in Mozambique (IRB00002657), on August 6, 2016. The ethical approvals were in consideration of Helsinki, as noted in the IRB approval letter. Written informed consent was obtained from all participants.”***

5. Please provide separate figure files in .tif or .eps format only, and remove any figures embedded in your manuscript file.  If you are using LaTeX, you do not need to remove embedded figures.

**Please find attached the following two tables in Tiff.**

***S1 Fig. Prevalence risk ratio of having someone in the household injured by a road traffic injury in the last 6 months***

**S2 Fig. Map of Prevalence Rate Ratio of Road Traffic Injuries in Sofala and Manica, Mozambique 2017**

6. Please provide a complete Data Availability Statement in the submission form, ensuring you include all necessary access information or a reason for why you are unable to make your data freely accessible. Note that it is not acceptable for the authors to be the sole named individuals responsible for ensuring data access.

PLOS defines a study's minimal data set as the underlying data used to reach the conclusions drawn in the manuscript and any additional data required to replicate the reported study findings in their entirety. Any potentially identifying patient information must be fully anonymized.

If your research concerns only data provided within your submission, please write ""All data are in the manuscript and/or supporting information files"" as your Data Availability Statement.

**We have deposited the fully anonymized data here**: <https://dataverse.harvard.edu/dataverse/incomas_rti>

7. Please amend your detailed Financial Disclosure statement. This is published with the article, therefore should be completed in full sentences and contain the exact wording you wish to be published.

i). State the initials, alongside each funding source, of each author to receive each grant.

**A financial disclosure is included in the manuscript (lines 284-288). It reads as follows: This study was supported by the Doris Duke Charitable Foundation’s African Health Initiative which was awarded to KS and FC, as co-Principal Investigators. The Doris Duke Charitable Foundation had no role in the design of the study, the collection, analysis, and interpretation of the data and in the writing of the manuscript.**

Besides, the manuscript need to be edited by a person with good knowledge of writing English for reader-friendliness.

**The manuscript has been edited by a person with good knowledge of writing English for reader friendliness.**

Reviewer's Responses to Questions

**Comments to the Author**

1. Does this manuscript meet PLOS Global Public Health’s [publication criteria](https://eur03.safelinks.protection.outlook.com/?url=https%3A%2F%2Fjournals.plos.org%2Fglobalpublichealth%2Fs%2Fcriteria-for-publication&data=04%7C01%7Caperaltasantos%40dgs.min-saude.pt%7Cd6e78db0eb174b26804808d97d53bbc7%7C22c84608f01d46c5802463cc962e5f51%7C0%7C0%7C637678620873933429%7CUnknown%7CTWFpbGZsb3d8eyJWIjoiMC4wLjAwMDAiLCJQIjoiV2luMzIiLCJBTiI6Ik1haWwiLCJXVCI6Mn0%3D%7C1000&sdata=zNSIjCRswUkIQR%2BD9Fx1mVY0mIb5nWq7bRKv%2BeWHr0s%3D&reserved=0)? Is the manuscript technically sound, and do the data support the conclusions? The manuscript must describe methodologically and ethically rigorous research with conclusions that are appropriately drawn based on the data presented.

Reviewer #1: Yes

Reviewer #2: Partly

2. Has the statistical analysis been performed appropriately and rigorously?

Reviewer #1: Yes

Reviewer #2: Yes

3. Have the authors made all data underlying the findings in their manuscript fully available (please refer to the Data Availability Statement at the start of the manuscript PDF file)?

The [PLOS Data policy](https://eur03.safelinks.protection.outlook.com/?url=https%3A%2F%2Fjournals.plos.org%2Fglobalpublichealth%2Fs%2Fdata-availability&data=04%7C01%7Caperaltasantos%40dgs.min-saude.pt%7Cd6e78db0eb174b26804808d97d53bbc7%7C22c84608f01d46c5802463cc962e5f51%7C0%7C0%7C637678620873943383%7CUnknown%7CTWFpbGZsb3d8eyJWIjoiMC4wLjAwMDAiLCJQIjoiV2luMzIiLCJBTiI6Ik1haWwiLCJXVCI6Mn0%3D%7C1000&sdata=HjyD7CM5%2FXag4JdN%2Fs5Mqp%2Bkr%2BRJFQwO2o4P0xVz5iU%3D&reserved=0) requires authors to make all data underlying the findings described in their manuscript fully available without restriction, with rare exception. The data should be provided as part of the manuscript or its supporting information, or deposited to a public repository. For example, in addition to summary statistics, the data points behind means, medians and variance measures should be available. If there are restrictions on publicly sharing data—e.g. participant privacy or use of data from a third party—those must be specified.

Reviewer #1: Yes

Reviewer #2: Yes

4. Is the manuscript presented in an intelligible fashion and written in standard English?

PLOS Global Public Health does not copyedit accepted manuscripts, so the language in submitted articles must be clear, correct, and unambiguous. Any typographical or grammatical errors should be corrected at revision, so please note any specific errors here.

Reviewer #1: Yes

Reviewer #2: Yes

Please find attached our responses to individual reviewer comments in **bold** below.

Reviewer 1

1. Appreciating authors for their initiative in the prevention of road traffic injuries.
   The authors mention they explored factors associated with RTIs in the central region of Mozambique and pinpointing geographical “hotspots” of RTI incidence. However, the title gives an impression about a magnitude study. The title of the survey can be modified.

**Response**: **We agree that the title points to a magnitude study, however we wanted to emphasize that RTI are often neglected and a significant cause of disability and mortality, while providing a compelling title to readers. To clarify that the study focuses on risk factors we changed the title to *“The neglected epidemic – Risk factors associated with road traffic injuries in Mozambique: Results of the 2016 INCOMAS study”.* (lines 1-2)**

2. In any injury prevention survey, operational definition is important. It is also essential to know who is the respondent and how the questions were asked. For Road traffic injury (RTI), what type of vehicle is considered both motorized and non-mortised? During asking the question, did the authors made sure whether respondents know what RTI is? Case definition needs to be more precise. For example, a person was pushed by a vehicle. Still, nothing happened, or persons could not perform their normal daily activities one day or three days or taken medicine from a pharmacy or be admitted in a hospital. On the other hand, some minor injuries were missed as the household head didn’t know it. According to the operational definition magnitude of the problem will be varied to a great extent. It will be better if the authors describe the methods in more detail, considering my concern.

**Response: Relevant case definitions and their sources have been clarified in the text (lines 103-113). The definitions of Road Traffic Accident and Road Traffic Injury were sourced from the World Health Organization (****<https://www.who.int/violence_injury_prevention/publications/road_traffic/world_report/glossary.pdf>). *Road traffic accident* is defined as “a collision involving at least one vehicle in motion on a public or private road that results in at least one person being injured or killed.” Road traffic accident and road vehicle accident are used interchangeably in our manuscript.**

**A *road traffic injury* is defined as ”fatal or non-fatal injuries incurred as a result of a road traffic crash.” Road traffic injury and road vehicle injury are used interchangeably in our manuscript.**

**We adapted our study definitions of minor and major injury (resulting from road traffic accident) from the government of the United Kingdom’s Department of Transport. (****<https://www.gov.uk/government/publications/road-accidents-and-safety-statistics-guidance/reported-road-casualties-in-great-britain-notes-definitions-symbols-and-conventions>). We defined resultant minor (or slight) injury as “a sustained injury which did not require hospitalization”, and major injury as “a sustained injury that required hospitalization”.**

Reviewer 2

1. Is it correct that the main question included in INCOMAS “Have any household members been injured as a result of any road vehicle accident in the last six months (either as an occupant or pedestrian)?” restricts answers to car occupants and pedestrians? If so, this is a highly restrictive (and v unusual) definition of a road traffic crash, which should include bicyclists, motorcyclists, bus occupants among others.

**Response: We clarified above (see response to previous reviewer’s comment) and in the manuscript (lines 103-113), the definition of road traffic accident. In addition, we clarified what types of transport were included in our use of vehicle - “veículo” in Portuguese. Specifically types of vehicles can include motorized (cars, buses, motorcycles, scooters, tuktuks) and non-motorized (rickshaws, carts and bicycles). Pedestrians included individuals walking, running or seated on the side of the road or in the road. (lines 116-122)**

2. The headline finding “6.12% of individuals reported an RTI in the last 6 months” (or 12% of the population annually) is a huge number. In most countries about 1-2% of the population are involved in non-fatal road traffic crashes (e.g. in the US, NHTSA reports 1.2 million injuries annually; WHO-GSRRS claims about 50 million injuries globally or less than 1%). So, does this survey show that the risks of road traffic injuries in Mozambique are much higher than in other LMICs? I suspect that this is not true and that most likely the injuries being reported in this survey are very minor injuries. Regardless, it is important that the paper discuss the findings of Mozambique in relation with estimates from other settings. We need some way to understand the numbers reported.

**Response: We included a clarification about the estimates in the manuscript, as we included both RTIs which resulted in both major (hospitalized, death) and minor (non-hospitalized) injuries. Most estimates in the literature emanate from emergency department data, thus only including major injuries (those requiring hospitalizations) resulting from RTI.**

**We have added this information in the manuscript in Table 1 and in lines 162-168, and 195-197.**

3. The methods section says that information on injury severity was collected)“We also collected the number of adults and children injured as a consequence of RTIs and the sequelae (injury, hospitalization, permanent disability ….”)). However, none of these results are presented in the manuscript. Note that these are commonly used to characterize injury severity and can be very useful for making sense of the very high estimates.

**Response: The Table 1 has been updated to include the sequelae of RTI data from our sample (number and proportion of total affected).**

4. I’m worried that the manuscript is not careful in how it cites past work. Examples:
   - The opening sentence of the introduction presents global estimates (“1.2 million deaths globally in 2015”) but cites references that all pre-date 2015. I recommend that the authors cite GBD-2019 and/or the WHO GSRRS 2018 for these statements.

**Response: That you for noting this. We have updated our data and citations to the most recent GBD (2019) and the WHO June 2021 fact sheet.**

5. The 3rd sentence claims that burden of RTI is worsening and that traffic injuries are expected to become the fifth leading cause of death, citing WHO’s 2015 GSRRS. However, GSRRS-2015 claims it will be the seventh (not fifth) leading cause.

**Response**: **That you for pointing this out. We updated the estimates with the WHO’s 2018 GSRRS**

6. Paragraph 2, Sentence 1 claims that road traffic crashes are decreasing in Mozambique. This is very unlikely to be true. I assume it is based on police reporting (the cited report is not accessible to me), which are known to severely under-report non-fatal crashes.

**Response**: **Indeed, this has been reworded to effectively convey that police registration of road traffic accidents has gone down from 2015 (2,848 reported accidents) to 2020 (1,038 reported accidents). More confusing is the variation across provinces with one province reporting a 100% increase in reported accidents in one year, and four other provinces reporting a greater than 40% reduction in the same period. This is all to say that the police reported data is unreliable. We also included the URL to the report from the National Statistics Institute of Mozambique in the endnote file.**

7. Injury severity need to be carefully described.
   - Paragraph 2, Sentence 3: “In Mozambique, nearly 25% of RTIs result in deaths”. This statement is not plausibly true. Consider that according to INCOMAS, 6% of the population has an RTI every year. With a case fatality rate of 0.25, the country would lose 1.5% of its population every year (or 7 million road traffic deaths every year).

**Response: Thanks for this point. This sentence has been omitted.**

8. The causes of road traffic crashes are inappropriately described.
   - E.g. Paragraph 2 in the Introduction says out that risk factors for RTI are “ poorly educated drivers and treacherous pedestrian behaviors”. Such language (which is victim blaming; and reflects class biases) should not be used. Similarly Paragraph 3 claims that “informal housing impairs adequate traffic signaling on roads”. There is no evidence that this is an issue.

**Response: Thanks for these comments. They have been removed as suggested by the reviewer.**

9. The discussion section concludes that there is a need to educate motorbike riders and to take away driving license for repeat offenders. This is an inappropriately narrow framing. There are a wide range of interventions available for reducing motorcyclist injuries.

**Response:** **We agree with the suggestion, and changed the sentence. We have also added into the future research and policy implications recommendations to improve the use of systematic traffic injury measurements to better help countries improve surveillance and contribute to RTI mitigation efforts globally.**

10. Many of the numbers in Paragraph 1 of Results are not consistent with Table 1.

**Response: These numbers have been crossed checked and corrected.**

6. PLOS authors have the option to publish the peer review history of their article ([what does this mean?](https://eur03.safelinks.protection.outlook.com/?url=https%3A%2F%2Fjournals.plos.org%2Fglobalpublichealth%2Fs%2Feditorial-and-peer-review-process%23loc-peer-review-history&data=04%7C01%7Caperaltasantos%40dgs.min-saude.pt%7Cd6e78db0eb174b26804808d97d53bbc7%7C22c84608f01d46c5802463cc962e5f51%7C0%7C0%7C637678620873943383%7CUnknown%7CTWFpbGZsb3d8eyJWIjoiMC4wLjAwMDAiLCJQIjoiV2luMzIiLCJBTiI6Ik1haWwiLCJXVCI6Mn0%3D%7C1000&sdata=aHUJw0wXsI6tskjtBwQISB32b0QWZF0TatQ56EfC0jw%3D&reserved=0)). If published, this will include your full peer review and any attached files.


**Do you want your identity to be public for this peer review?** If you choose “no”, your identity will remain anonymous but your review may still be made public.


For information about this choice, including consent withdrawal, please see our [Privacy Policy](https://eur03.safelinks.protection.outlook.com/?url=https%3A%2F%2Fwww.plos.org%2Fprivacy-policy&data=04%7C01%7Caperaltasantos%40dgs.min-saude.pt%7Cd6e78db0eb174b26804808d97d53bbc7%7C22c84608f01d46c5802463cc962e5f51%7C0%7C0%7C637678620873953338%7CUnknown%7CTWFpbGZsb3d8eyJWIjoiMC4wLjAwMDAiLCJQIjoiV2luMzIiLCJBTiI6Ik1haWwiLCJXVCI6Mn0%3D%7C1000&sdata=QcA6De%2FFtkIZOiN3Gh1I92a%2FAKwdgknskOv%2FAIDuA5Y%3D&reserved=0).

Reviewer #1: **Yes:** Saidur Rahman Mashreky

Reviewer #2: **Yes:** Kavi Bhalla

[NOTE: If reviewer comments were submitted as an attachment file, they will be attached to this email and accessible via the submission site. Please log into your account, locate the manuscript record, and check for the action link "View Attachments". If this link does not appear, there are no attachment files.]

While revising your submission, please upload your figure files to the Preflight Analysis and Conversion Engine (PACE) digital diagnostic tool, [https://pacev2.apexcovantage.com/](https://eur03.safelinks.protection.outlook.com/?url=https%3A%2F%2Fpacev2.apexcovantage.com%2F&data=04%7C01%7Caperaltasantos%40dgs.min-saude.pt%7Cd6e78db0eb174b26804808d97d53bbc7%7C22c84608f01d46c5802463cc962e5f51%7C0%7C0%7C637678620873953338%7CUnknown%7CTWFpbGZsb3d8eyJWIjoiMC4wLjAwMDAiLCJQIjoiV2luMzIiLCJBTiI6Ik1haWwiLCJXVCI6Mn0%3D%7C1000&sdata=N38mXjoe%2F8N5itAvWMGt%2FityS%2BHDlwxcyxNxsd%2B2egw%3D&reserved=0). PACE helps ensure that figures meet PLOS requirements. To use PACE, you must first register as a user. Registration is free. Then, login and navigate to the UPLOAD tab, where you will find detailed instructions on how to use the tool. If you encounter any issues or have any questions when using PACE, please email PLOS at [figures@plos.org](mailto:figures@plos.org). Please note that Supporting Information files do not need this step.
